# Socio-Demographic Disparities in Diet and Their Association with Physical and Mental Well-Being: Million-Participant Cross-Sectional Study in Poland

**DOI:** 10.3390/nu17182924

**Published:** 2025-09-11

**Authors:** Grażyna Zweifler, Anna Zimny-Zając, Mateusz Babicki, Karolina Kłoda, Grzegorz Mazur, Beata Jankowska-Polańska, Agnieszka Mastalerz-Migas, Siddarth Agrawal

**Affiliations:** 1 Polish Society of Family Medicine, ul. Syrokomli 1, 51-141 Wroclaw, Poland; grazyna.zweifler@gmail.com; 2 Medonet, Ringier Axel Springer Poland, Domaniewska St. 49, 02-672 Warsaw, Poland; anna.zimny-zajac@medonet.pl; 3 Department of Family Medicine, Faculty of Medicine, Wroclaw Medical University, 50-367 Wroclaw, Poland; agnieszka.mastalerz-migas@umw.edu.pl; 4 MEDFIT Karolina Kloda, 70-240 Szczecin, Poland; 5 Department of Emergency Medical Service, Wroclaw Medical University, 51-616 Wroclaw, Poland; grzegorz.mazur@umw.edu.pl; 6 Faculty of Medicine, Wroclaw University of Science and Technology, 50-425 Wroclaw, Poland; bpolanska@4wsk.pl; 7 Labplus R&D, Wyspa Slodowa 7, 50-266 Wroclaw, Poland; siddarth@agrawal.pl

**Keywords:** dietary habits, public health, mental health, health disparities, Poland, socio-demographic factors, self-rated health nutrition, well-being

## Abstract

Background: Dietary habits are key determinants of physical and mental health, yet large-scale, contemporary data on these behaviors and their health correlates are crucial for effective public health policy. This study leverages a uniquely large dataset to quantify the eating behaviors of Polish adults and examines how these behaviors relate to socio-demographic characteristics, health status, and subjective well-being. Methods: We analyzed data from 1,196,102 adult respondents who participated in the National Poles’ Health Test, a recurring online survey, between 2019 and 2024. The study used self-reported data on dietary habits, socio-demographic variables, chronic conditions, and self-assessed physical and mental health. Descriptive statistics and comparative analyses, such as the chi-square test, Student’s *t*-test, or ANOVA, were used. Results: Our findings reveal stark socio-demographic disparities in nutrition. Younger respondents and lower educational attainment were strongly correlated with more frequent consumption of fast food and sugar-sweetened beverages. Crucially, these unhealthy eating patterns were significantly associated with poorer self-rated physical and, notably, mental health (among people who consumed fast food every day, as many as 16.6% rated their mental health as bad, and 6.7% as very bad). In contrast, higher vegetable and fruit consumption correlated with markedly better health outcomes and greater subjective well-being. Conclusions: This large-scale study provides evidence of the profound link between diet and both physical and mental well-being in Poland. The results underscore the urgent need for targeted public health strategies aimed at improving dietary behaviors, particularly among younger and less-educated populations. The strong associations between diet, health status, and well-being highlight the importance of integrating nutritional education into both general healthcare and mental health services.

## 1. Introduction

Poor dietary choices are a leading global risk factor for chronic disease and premature mortality. In response, nutrition experts continually develop dietary guidelines aimed at supporting individuals in making informed food choices that promote both physical and mental health. While generally emphasizing balanced nutrition and physical activity, these guidelines are often tailored to individual factors such as age, gender, type of employment, preferred physical activity, and comorbid health conditions [1,2,3]. The challenge lies in influencing eating habits, which begin forming in early childhood, shaped by a complex interplay of the immediate environment, peer influence, socioeconomic status, and access to specific food products [4,5,6]. Proper nutrition provides the body with essential nutrients—including carbohydrates, fats, proteins, and vitamins—necessary for healthy psychophysical development [1,2,3]. A well-designed diet can not only alleviate the symptoms of various diseases but, in some cases, contribute to their complete remission. Conversely, poor dietary choices often result in irreversible health consequences, leading to disability or premature death [7,8,9,10].

In the Polish context, while malnutrition encompasses both insufficient or excessive caloric intake, the latter presents a more widespread public health challenge. Although hunger is not a widespread issue in Poland, caloric deficiencies are typically observed among individuals with mental disorders (e.g., anorexia, depression) or somatic illnesses such as cancer [11,12,13]. More commonly, however, the growing trend involves excessive consumption, particularly of highly processed, sugar-laden products. This trend is a major contributor to the rise of so-called “civilization diseases” such as obesity, type 2 diabetes mellitus, hypertension, and depression [14,15]. To address this, Polish public health professionals continue to make efforts to raise awareness and facilitate healthy food choices, with educational programs targeting a wide range of audiences from preschool children to older adults. As a member of the European Union, Poland is obligated to comply with EU directives aimed at combating the global obesity epidemic. Measures have included the implementation of a sugar tax on sweetened beverages to discourage excessive consumption and the promotion of mobile applications that allow consumers to easily evaluate the composition and origin of food products [16,17,18,19]. However, the impact of some policies has been inconsistent; for example, an initiative to remove unhealthy snacks from school vending machines was not sustained long-term.

Nevertheless, despite a slight improvement in nutritional awareness, Poland still lags behind more developed nations. The key challenges include insufficient education on healthy eating, limited access to qualified nutritionists, especially within the public healthcare system, as well as stigma and reluctance among individuals to seek professional help. Crucially, the design and targeting of these public health strategies are impeded by a lack of large-scale, contemporary data on the specific dietary patterns prevalent across diverse socio-demographic segments of the Polish population. This evidence gap is particularly relevant as the healthcare landscape evolves; novel changes within the primary healthcare in Poland are implementing coordinated care and a new prevention program ‘My Health,’ both of which provide access to dietary consultations as healthcare interventions improving disease treatment or as preventive measures [20,21,22,23].

Therefore, the aim of this study was to address this evidence gap by leveraging the uniquely large dataset of the National Test of Poles’ Health. We sought to provide a comprehensive, data-driven analysis of nutritional patterns among Polish adults, identify key socio-demographic disparities, and quantify the association between these habits and self-rated physical and mental health. The recurring nature of the test allows for longitudinal tracking of Poles’ health status and provides insight into the effectiveness of public health initiatives aimed at improving nutritional awareness. The main hypothesis of this study was that the eating habits of Poles are diverse and related to socio-demographic factors (age, gender, education, place of residence), level of health education, as well as affect health status and its perception.

## 2. Materials and Methods

### 2.1. Study Design and Population

This study employed a cross-sectional design, analyzing data collected from the National Poles’ Health Test, a recurring online survey conducted between 2019 and 2024.The questionnaire, administered in Polish, was published online through Medonet, a major health-related digital platform, and disseminated via national media, social networking sites, and targeted advertisements to achieve broad reach across diverse demographic groups. The study included adult (≥18 years old) Polish internet users who voluntarily participated; no other specific exclusion criteria were applied. Over the five-year period, a total of 1,196,102 respondents were surveyed. The study used convenience sampling through voluntary online participation. No formal sample size calculation was performed as the aim was to achieve the largest possible sample through the available platform. The study was designed to analyze the eating habits of Poles and their impact on health by evaluating dominant dietary patterns, identifying influencing socio-demographic and psychological factors, and assessing the relationship between diet, chronic diseases, and subjective health status. The reporting of this study adheres to the Strengthening the Reporting of Observational Studies in Epidemiology (STROBE) guidelines for cross-sectional studies (Supplementary Materials Table S1). The main hypothesis was that eating habits are diverse and related to socio-demographic factors (age, gender, education, place of residence) and health education, which in turn affect health status and its perception. Specific hypotheses proposed that younger people would be more likely to consume unbalanced diets, including fast food, than older people; that higher education would correlate with more balanced dietary choices; that individuals following a balanced diet would report better health and fewer chronic diseases; and that stress would influence food choices, leading to consumption of unhealthy snacks.

### 2.2. Survey and Data Collection, and Ethical Consideration

The survey instrument was developed by a team of public health specialists and survey methodologists from Medonet. Prior to its nationwide launch, the questionnaire underwent pilot testing with a sample of 100 adult internet users to assess clarity, comprehensibility, and technical functionality, with feedback used to refine the final version. The questionnaire collected data across several key domains. Socio-demographic information included participants’ age, sex, place of residence (degree of urbanization), and highest level of education. Eating habits were assessed through a series of questions regarding the type of diet followed (e.g., balanced, vegetarian, meat-based), the frequency of consuming fast food (hamburgers, fries), sugar-sweetened beverages, and energy drinks. Consumption of healthy foods was measured by the frequency of eating vegetables and fruits. Additional behavioral questions explored adherence to weight loss diets and eating behaviors in stressful situations. Health status was evaluated based on self-reported height and weight to calculate Body Mass Index (BMI) and the presence of any physician-diagnosed chronic diseases.

The study was conducted in accordance with the ethical standards of the Declaration of Helsinki. Participation was fully anonymous and voluntary, with no incentives provided. All participants provided informed consent online before beginning the survey, after being briefed on the study’s objectives. The Bioethics Committee of the Military Chamber of Physicians reviewed the study proposal and determined that no formal ethical approval was required (Decision No. KB 65/2024). Data were fully anonymized to ensure participant confidentiality and compliance with data protection regulations. For a better understanding of the research methodology, the English version of the survey was attached in Supplementary Table S2 (the English version of the study questionnaire).

### 2.3. Statistical Analysis

Data were analyzed using a multi-step approach. First, descriptive statistics, including frequencies, measures of central tendency (mean, median), and dispersion (standard deviation), were calculated to summarize participant characteristics and dietary habits. Next, comparative analyses, such as the chi-square test, Student’s *t*-test, or ANOVA, were used to test for significant differences in eating habits and health status between socio-demographic groups. Complete case analysis was used for all statistical tests. Participants with missing data for specific variables were excluded from relevant analyses. No sensitivity analyses were performed given the descriptive nature of the study. The choice between parametric (e.g., Student’s *t*-test, ANOVA) and non-parametric tests (e.g., Mann–Whitney U test) was based on the nature of the data and fulfillment of test assumptions. All statistical analyses were conducted using Statistica 13.3 (StatSoft, Inc., Tulsa, OK, USA).

## 3. Results

### 3.1. Participant Characteristics

In total, 1,196,102 participants took part in the study, with a slight predominance of women (n = 619,294; 51.77). The mean age of participants was 44.7 ± 15.3 years, with the largest age group being those aged 55 and older (26.9%). A substantial portion of respondents were rural residents (34.5%), and 37.2% of participants had higher education. Nearly half of all respondents (49.8%) reported having at least 1 chronic disease, with hypertension (28.0%) and asthma/allergy (20.6%) being the most common. The detailed characteristics of the study group are presented in Table 1.

### 3.2. Assessment of Eating Habits

When asked about their diet, the most frequent response was “I don’t know/it’s hard to say” (32.6%). Among those who did declare a specific diet, a balanced diet was indicated most often. In assessing individual food items, 31.9% of respondents reported consuming vegetables every day, and 35.0% ate fruit every day. The study group declared a high consumption of sweetened beverages, with 31.3% of respondents declaring their consumption at least once a week, with 11.7% consuming them every day. For fast food consumption, the most common answer was less than once a month (40.4%); however, 8.5% of respondents admitted to consuming this type of food at least once a week. A detailed summary of dietary habits is presented in Table 2.

### 3.3. Association of Dietary Habits with Self-Rated Health

Dietary habits demonstrated a strong association with participants’ self-assessment of both their physical and mental health. Regarding mental health, it was shown that the more often respondents ate fast food, the lower they rated their mental health. For example, among people who consumed fast food every day, as many as 16.6% rated their mental health as bad, and 6.7% as very bad. Similar negative associations were observed for the consumption of energy drinks and sweetened beverages. Conversely, a higher frequency of fruit and vegetable consumption was associated with a higher assessment of mental health. A detailed summary is presented in Table 3.

A similar relationship was found with physical health, where unhealthy eating habits were associated with a poorer self-assessment. For example, individuals who consumed fast food or energy drinks every day were more likely to rate their physical health as poor or very poor. In contrast, daily consumption of fruit and vegetables was strongly associated with positive self-assessments, with these individuals most often indicating that their physical health was ‘very good’. A detailed summary is presented in Table 4.

### 3.4. Associations with Socio-Demographic, Psychosocial, and Educational Factors

**Figure 1:**
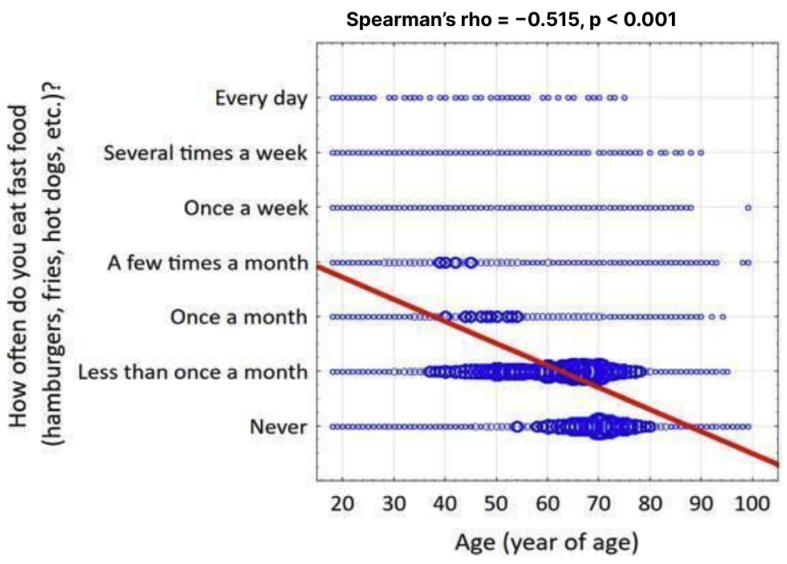
Correlation of fast food consumption frequency depending on age and the Spearman correlation coefficient rho.

The results confirmed associations between dietary choices and various socio-demographic and psychosocial factors, aligning with several of the study’s initial hypotheses. key finding was that younger respondents were more likely to choose unbalanced diets, including fast food, than older respondents. This was supported by a significant negative correlation between the frequency of fast food consumption and the age of the respondents (Spearman’s rho = −0.515, *p* < 0.001), with findings detailed in Figure 1. However, the hypothesis that people living in cities would choose healthier diets was not confirmed. No significant difference was found for balanced or vegetarian diets based on place of residence. Specifically, the use of a balanced diet was not related to urbanization, as the proportion of people following one in the countryside (19.3%) did not differ significantly from those in cities with over half a million inhabitants (19.0%).

**Figure 2:**
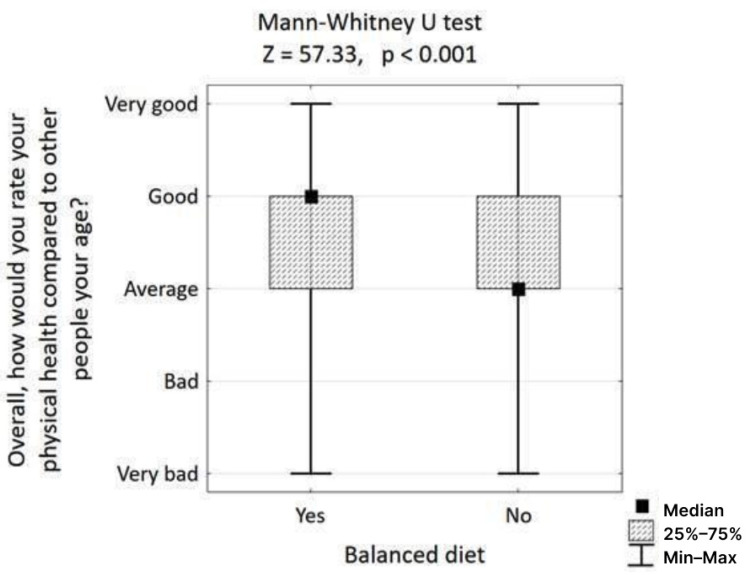
Association of physical health with choosing a balanced diet.

**Figure 3:**
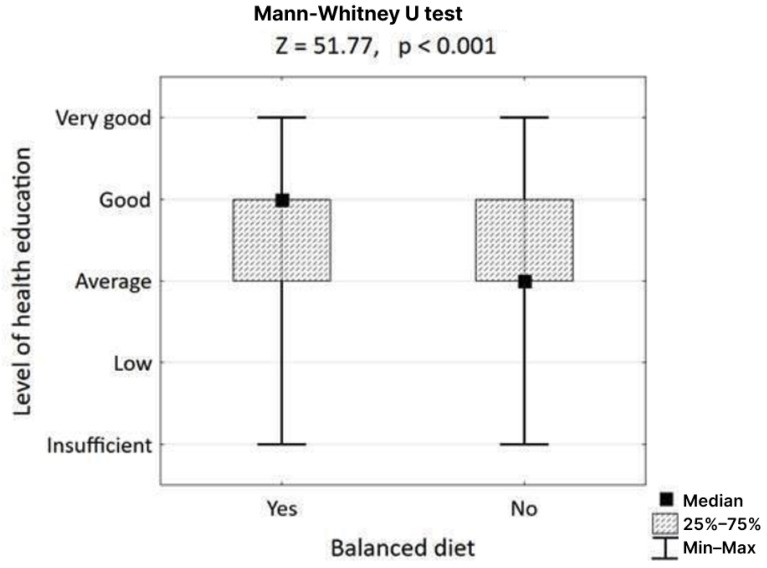
Association of health level education with choosing a balanced diet.

In contrast, adherence to a balanced diet was strongly associated with health outcomes and education. The data confirmed that people who follow a balanced diet rate their physical health significantly better (Mann–Whitney U test, Z = 57.33, *p* < 0.001) than those who do not (Figure 2). Furthermore, the hypothesis that a higher level of health education correlates with better dietary choices was supported; individuals following a balanced diet reported a significantly higher level of acquired health education (Mann–Whitney U test, Z = 51.77, *p* < 0.001) (Figure 3).

**Figure 4:**
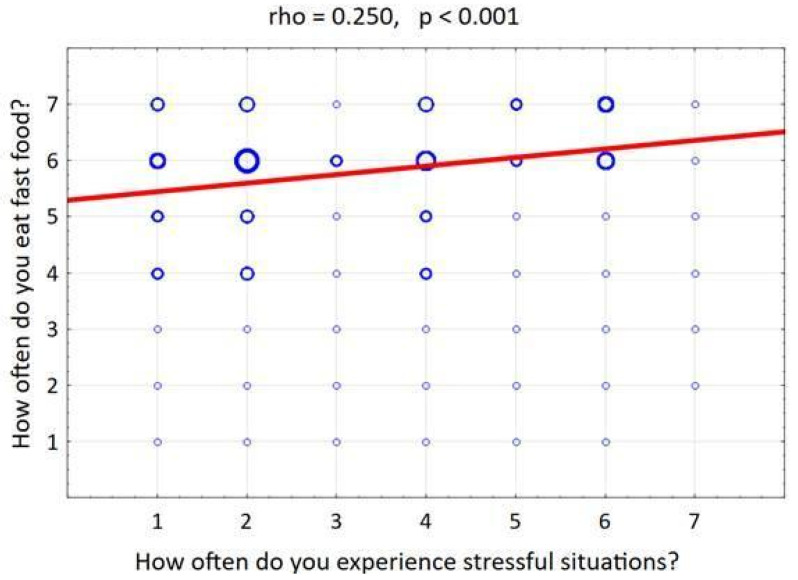
Relationship between perceived stress and fast-food consumption frequency. Blue circles denote observation clusters; circle area is proportional to the number of participants in each stress–consumption combination. The red line depicts a fitted linear trend; association is summarized by Spearman’s ρ = 0.250, *p* < 0.001.

Finally, the results supported the hypothesis that stress affects food choices, leading to reaching for unhealthy options. A positive correlation was observed between the frequency of experiencing stressful situations and the frequency of consuming fast food (rho = 0.250, *p* < 0.001), as shown in Figure 4.

## 4. Discussion

This study, based on a uniquely large dataset of over one million Polish adults, provides evidence that dietary habits are strongly associated with socio-demographic factors and self-rated health. Our analysis confirmed several initial hypotheses, revealing that younger age and lower health education are linked to poorer dietary choices, while stress is correlated with increased consumption of fast food. Crucially, we quantified a significant dose–response relationship between the consumption of unhealthy foods and poorer physical and mental health outcomes. Interestingly, our findings did not support the common assumption that urban living is associated with healthier dietary patterns compared to rural settings, which, in Poland, are characterized with higher obesity prevalence indicated by the Polish Parliamentary Analysis Office.

The confirmation that younger respondents report more frequent consumption of fast food and other unbalanced dietary options aligns with global data indicating a trend toward convenience-oriented diets among youth [24]. This finding highlights the powerful influence of the modern “obesogenic environment,” where unhealthy food is often highly accessible and affordable, contributing to weight gain and its adverse health consequences [25]. In stark contrast, the high participation rate of individuals in the 55+ age group (26.9%) suggests that with age and the onset of health concerns, awareness of the importance of proper lifestyle habits increases, motivating individuals to seek health-related information and assessment [26].

A key contribution of this study is the clarification of the roles of education and geography in shaping dietary choices. Individuals with higher health education levels were more likely to follow a balanced diet, a trend similarly observed in other studies [27]. Participants with higher levels of education—a proxy for health literacy—were more likely to choose balanced diets and less likely to consume ultra-processed foods. This suggests that nutritional awareness and critical thinking about food choices are essential components of preventive health behavior. The hypothesis that has not been confirmed was that people living in cities choose a balanced diet more frequently than people living in the countryside. This observation is in agreement with data from other European countries. Analysis of cross-sectional data from the European Survey of Living Conditions revealed that health status is associated mainly with education—the higher the education, the lower the probability of poor health and with the household arrangements. Living with a partner is associated with a lower probability of poor health. Thus, these factors have greater influence than place of residence [5].

According to the data collected, Poles do not perform poorly in the context of global dietary trends. In fact, the COVID-19 pandemic marked a turning point, prompting many individuals to reflect on their health. Limited access to healthcare services during this time led people to seek alternative, often internet-based, methods for improving their health—beginning with dietary changes. With restaurants closed, meals were increasingly prepared at home, resulting in greater attention being paid to the quality and composition of purchased foods [28,29,30]. However, Poland is still lacking in multi-level nutrition education and health promotion. These are public health challenges, which are difficult to implement also in other countries [31,32]. Notably, over 30% of respondents consumed sweetened beverages at least once a week, and nearly 9% consumed fast food at least once a week, indicating a significant public health concern given the known links between such dietary habits and cardiometabolic risk [33,34,35]. Moreover, nearly half of the respondents reported at least one chronic disease, with hypertension and asthma/allergy being the most prevalent. The prevalence of overweight and obesity (60.6% combined) aligns with national statistics, which are constantly increasing, especially among children and adolescents and reinforces the need for targeted interventions [36].

Perhaps the most critical finding is the strong, dose-dependent association between dietary patterns and self-rated mental health, demonstrated here on an unprecedented scale. Individuals consuming fast food, energy drinks, and sweetened beverages more frequently were significantly more likely to report poorer mental health, while those consuming vegetables and fruits daily reported better mental well-being, a finding consistent with a growing body of evidence linking poor diet quality to adverse psychological outcomes through mechanisms like inflammation, gut microbiota disruption, and glycemic variability [37,38,39]. Notably, the proportion of respondents reporting poor mental health was highest among those who consumed fast food daily, supporting the hypothesis that stress and poor nutrition may act in a cyclical, mutually reinforcing manner. This supports other Polish studies that have demonstrated associations between higher BMI and more severe symptoms of anxiety and depression, reinforcing the deep connection between metabolic and mental health [40].

Our findings demonstrate a strong association between dietary behaviors and self-reported physical health among Polish adults. The results confirm that consumption of nutrient-poor, energy-dense foods such as fast food, sweetened beverages, and energy drinks is negatively related to the perceived quality of physical health, while frequent intake of fruits and vegetables is associated with more favorable health assessments. This observation supports a substantial body of evidence linking fruit and vegetable consumption with lower incidence of chronic diseases and improved physical functioning [41,42].

This study’s major strength lies in its unprecedented sample size and the breadth of data collected across multiple years, allowing for robust subgroup analyses not visible in smaller-scale studies. However, some limitations must be noted. First, the data were collected through a self-administered online survey, which may introduce selection bias toward more digitally literate individuals and/or those more motivated to participate, thus being not representative for the whole population, potentially leading to an overestimation of healthy habits and better self-rated health in our sample. Second, self-reported dietary behaviors and health outcomes may be subject to recall and social desirability bias such as inadequate reporting of comorbidities, which could weaken the observed associations by underestimating the consumption of unhealthy foods. Third, the cross-sectional design limits causal inference; while associations are strong and plausible, temporal ordering cannot be established. Additionally, the dietary classifications were based on participants’ self-reports (e.g., ‘balanced diet’), which is a practical approach for a large-scale survey but may lack the precision of formal dietary pattern analysis. Finally, while our analysis identified key relationships, the complexity of dietary patterns warrants more advanced modeling in future work.

## 5. Conclusions

This nationwide study shows that dietary patterns in Poland are systematically shaped by socio-demographic factors such as age, education, income, and place of residence. These patterns are consistently linked with both physical health status and psychological well-being, highlighting diet as a key pathway connecting social position and overall health. By demonstrating these dual associations, our work extends existing evidence and provides a strong rationale for integrating dietary counseling into both chronic disease management and mental healthcare.

## Figures and Tables

**Table 1:** Characteristics of the study group.

Variable		Total N (%)
Sex	Women	619,294 (51.77%)
	Men	576,808 (48.23%)
Age (years) M ± SD		44.7 ± 15.3
Age	18–24	114,128 (9.5%)
	25–34	236,976 (19.8%)
	35–44	293,433 (24.5%)
	45–54	229,648 (19.2%)
	55+	321,918 (26.9%)
Place of residence	Village	413,166 (34.5%)
	Up to 19 k inhab.	142,540 (11.9%)
	20–49 k inhab.	153,755 (12.9%)
	50–99 k inhab.	119,493 (10.0%)
	100–199 k inhab.	113,578 (9.5%)
	200–499 k inhab.	104,995 (8.8%)
	500+ k inhab.	148,574 (12.4%)
Education level	Basic	309,560 (25.9%)
	Secondary	441,549 (36.9%)
	Higher	444,992 (37.2%)
Chronic diseases	Any chronic disease	
	Arterial hypertension	334,367 (28.0%)
	Diabetes mellitus	92,451 (7.7%)
	Heart disease	128,515 (10.7%)
	COPD	27,423 (2.3%)
	Asthma/allergy	245,995 (20.6%)
	Depression	159,778 (13.4%)
	Cancer	51,954 (4.3%)
	Joint disease	213,222 (17.8%)
	Neurological disease	127,767 (10.7%)
BMI	>30	256,098 (21.4%)
	25–29.9	421,429 (39.2%)
	18.5–24.9	481,839 (40.2%)
	<18.5	24,771 (2.00%)

Missing data rates were <2% for all variables. COPD—Chronic Obstructive Pulmonary Disease.

**Table 2:** Presentation of eating habits in the study group.

Variable		N (%)
Being on diet	Balanced	254,976 (21.3%)
	Vegetarian	54,403 (4.5%)
	Vegan	5709 (0.5%)
	Meat	324,763 (27.2%)
	Gluten free	9228 (0.8%)
	Dairy free	8520 (0.7%)
	With carbohydrate restriction	47,782 (4.0%)
	With less sodium	72,095 (6.0%)
	Other type of meals	28,334 (2.4%)
	I don’t know/it’s hard to say	390,291 (32.6%)
Eating fast food?	Every day	2965 (0.2%)
	Several times a week	31,699 (2.7%)
	Once a week	66,498 (5.6%)
	A few times a month	224,699 (18.8%)
	Once a month	203,510 (17.0%)
	Less than once a month	482,696 (40.4%)
	I never eat such products	184,035(15.4%)
Drinking sweetened beverages	Every day	139,778 (11.7%)
	Several times a week	154,317 (12.9%)
	Once a week	80,241 (6.7%)
	A few times a month	201,909 (16.9%)
	Once a month	95,931 (8.0%)
	Less than once a month	293,452 (24.5%)
	I never drink such products	230,475 (19.3%)
Energy drinks	3 or more times a day	4691 (0.4%)
	1 to 2 times a day	13,059 (1.1%)
	Several times a week	36,766 (3.1%)
	Once a week	24,057 (2.0%)
	A few times a month	62,056 (5.2%)
	Once a month	47,126 (3.9%)
	Less than once a month	201,997 (16.9%)
	I never drink such drinks	806,349 (67.4%)
Eating Vegetables	Every day	381,998 (31.9%)
	Several times a week	509,451 (42.6%)
	Once a week	109,104 (9.1%)
	A few times a month	137,607 (11.5%)
	Once a month	23,286 (1.9%)
	Less than once a month	28,887 (2.4%)
	I never eat vegetables	5769 (0.5%)
Eating Fruits	Every day	418,973 (35.0%)
	Several times a week	455,568 (38.1%)
	Once a week	113,601 (9.5%)
	A few times a month	136,884 (11.4%)
	Once a month	28,473 (2.4%)
	Less than once a month	35,160 (2.9%)
	I never eat fruit	7444 (0.6%)

**Table 3:** Number of respondents in subgroups depending on the declared frequencies of consumed products and mental health.

Variable		Mental Health					
		Very Good	Good	Average	Poor	Very Poor	*p* ^1^
Fast food	Every day	500 (16.8%)	703 (23.7%)	756 (25.5%)	626 (21.1%)	383 (12.9%)	<0.001
	Several times a week	4242 (13.4%)	9086 (28.7%)	10,432 (32.9%)	6276 (19.8%)	1666 (5.3%)	
	Once a week	10,177 (15.3%)	23,932 (36.0%)	20,782 (31.3%)	9735 (14.6%)	1874 (2.8%)	
	A few times a month	38,210 (17.0%)	84,269 (37.5%)	68,346 (30.4%)	28,874 (12.8%)	5002 (2.2%)	
	Once a month	40,158 (19.7%)	83,433 (41.0%)	56,130 (27.6%)	20,578 (10.1%)	3213 (1.6%)	
	Less than once a month	111,235 (23.0%)	205,849 (42.6%)	121,007 (25.1%)	38,801 (8.0%)	5807 (1.2%)	
	Never	53,885 (29.3%)	79,391 (43.1%)	38,496 (20.9%)	10,647 (5.8%)	1618 (0.9%)	
Drinking beverages	Every day	26,731 (19.1%)	53,280 (38.1%)	39,641 (28.4%)	16,393 (11.7%)	3735 (2.7%)	<0.001
	Several times a week	28,029 (18.2%)	60,264 (39.1%)	43,971 (28.5%)	18,494 (12.0%)	3561 (2.3%)	
	Once a week	15,339 (19.1%)	32,527 (40.5%)	22,555 (28.1%)	8392 (10.5%)	1431 (1.8%)	
	A few times a month	39,167 (19.4%)	82,014 (40.6%)	56,428 (27.9%)	21,027 (10.4%)	3275 (1.6%)	
	Once a month	19,156 (20.0%)	39,568 (41.2%)	26,416 (27.5%)	9431 (9.8%)	1363 (1.4%)	
	Less than once a month	66,537 (22.7%)	124,404 (42.4%)	74,446 (25.4%)	24,606 (8.4%)	3461 (1.2%)	
	I never drink such drinks	63,448 (27.5%)	94,607 (41.0%)	52,490 (22.8%)	17,195 (7.5%)	2737 (1.2%)	
Energy drinks	Three or more times a day	803 (17.1%)	1082 (23.1%)	1377 (29.3%)	1025 (21.8%)	407 (8.7%)	<0.001
	One to two times a day	1912 (14.6%)	3778 (28.9%)	4144 (31.7)	2414 (18.5%)	813 (6.2%)	
	Several times a week	6021 (16.4)	11,572 (31.5)	11,339 (30.8)	6298 (17.1%)	1538 (4.2%)	
	Once a week	4399 (18.3%)	8652 (36.0%)	6974 (29.0%)	3276 (13.6%)	759 (3.2)	
	A few times a month	10,829 (17.4%)	22,672 (36.5%)	18,166 (29.3%)	8516 (13.7%)	1875 (3.0%)	
	Once a month	8409 (17.8%)	18,329 (38.9%)	13,466 (28.6%)	5788 (12.3)	1136 (2.4%)	
	Less than once a month	40,673 (20.1%)	81,701 (40.4%)	55,128 (27.3%)	21,088 (10.4%)	3410 (1.7)	
	I never drink energy drinks	185,361 (23.0%)	338,879 (42.0%)	205,352 (25.5%)	67,133 (8.3%)	9626 (1.2%)	
Drinking alcohol	Two or more drinks a day	5476 (17.0%)	11,047 (34.3%)	9750 (30.3%)	4753 (14.8%)	1143 (3.6%)	<0.001
	About one drink a day	9291 (21.4%)	17,676 (40.7%)	11,637 (26.8%)	4180 (9.6%)	691 (1.6%)	
	Two or three drinks a week	35,224 (21.9%)	66,469 (41.2%)	42,504 (26.4%)	14,832 (9.2%)	2140 (1.3%)	
	Two or three drinks a month	39,030 (21.3%)	75,255 (41.1%)	48,506 (26.5%)	17,476 (9.5%)	2746 (1.5%)	
	One drink a month or less	48,543 (20.6%)	95,921 (40.6%)	64,679 (27.4%)	23,241 (9.8%)	3780 (1.6%)	
	I never drink alcohol	33,193 (20.2%)	59,965 (36.6%)	46,221 (28.2%)	20,202 (12.3%)	4441 (2.7%)	
Vegetables	Every day	104,985 (40.6%)	154,086 (31.7%)	87,439 (27.7%)	30,626 (26.5%)	4862 (24.9%)	<0.001
	Several times a week	105,698 (40.9%)	214,361 (44.0%)	134,492 (42.6%)	47,509 (41.1%)	7391 (37.8%)	
	Once a week	17,652 (6.8%)	43,809 (9.0%)	33,015 (10.4%)	12,588 (10.9%)	2039 (10.4%)	
	A few times a month	21,588 (8.4%)	54,521 (11.2%)	42,095 (13.3%)	16,328 (14.1%)	3075 (15.7%)	
	Once a month	3229 (1.2%)	8540 (1.8%)	7576 (2.4%)	3291 (2.8%)	652 (3.3%)	
	Less than once a month	4149 (1.6%)	9751 (2.0%)	9625 (3.0%)	4296 (3.7%)	1067 (5.5%)	
	Never	1103 (0.4%)	1592 (0.3%)	1703 (0.5%)	898 (0.8%)	473 (2.4%)	
Fruit	Every day	112,967 (43.7%)	179,472 (36.9%)	93,961 (29.7%)	28,604 (24.8%)	3969 (20.3%)	<0.001
	Several times a week	93,923 (36.3%)	190,099 (39.1%)	122,009 (38.6%)	42,830 (37.1%)	6707 (34.3%)	
	Once a week	18,812 (7.3%)	43,410 (8.9%)	34,420 (10.9%)	14,400 (12.5%)	2559 (13.1%)	
	A few times a month	21,917 (8.5%)	50,751 (10.4%)	42,719 (13.5%)	18,228 (15.8%)	3270 (16.7%)	
	Once a month	4163 (1.6%)	9635 (2.0%)	9388 (3.0%)	4308 (3.7%)	979 (5.0%)	
	Less than once a month	5129 (2.0%)	11,286 (2.3%)	11,333 (3.6%)	5852 (5.1%)	1559 (8.0%)	
	Never	1492 (0.6%)	2009 (0.4%)	2114 (0.7%)	1312 (1.1%)	516 (2.6%)	
Red meat	Every day	8913 (20.7%)	16,326 (38.0%)	12,215 (28.4%)	4629 (10.8%)	905 (2.1%)	<0.001
	One to three times a week	66,108 (21.5%)	127,695 (41.5%)	81,893 (26.6%)	27,701 (9.0%)	4261 (1.4%)	
	Once or twice a month	74,535 (21.1%)	141,676 (40.2%)	94,887 (26.9%)	35,498 (10.1%)	5952 (1.7%)	
	Never	12,351 (17.0%)	26,164 (36.0%)	22,184 (30.5%)	9677 (13.3%)	2256 (3.1%)	

^1^ *p*-value derived from the chi-square test for independence.

**Table 4:** Number of respondents in subgroups depending on the declared frequencies of consumed products and physical health.

Variable		Physical Health					
		Very Good	Good	Average	Poor	Very Poor	*p* ^1^
Fast food	Every day	488 (16.4%)	638 (21.5%)	1149 (38.7%)	494 (16.6%)	198 (6.7%)	<0.001
	Several times a week	3145 (9.9%)	9730 (30.7%)	13,980 (44.1%)	4212 (13.3%)	633 (2.0%)	
	Once a week	7941 (11.9%)	25,594 (38.5%)	26,733 (40.2%)	5660 (8.5%)	573 (0.9%)	
	A few times a month	26,936 (12.0%)	89,590 (39.9%)	89,598 (39.9%)	16,910 (7.5%)	1667 (0.7%)	
	Once a month	27,987 (13.8%)	85,948 (42.2%)	75,218 (37.0%)	13,090 (6.4%)	1270 (0.6%)	
	Less than once a month	70,430 (14.6%)	202,050 (41.9%)	174,976 (36.2%)	32,090 (6.6%)	3152 (0.7%)	
	Never	31,686 (17.2%)	75,607 (41.1%)	62,346 (33.9%)	12,788 (6.9%)	1609 (0.9%)	
Drinking beverages	Every day	14,407 (10.3%)	49,380 (35.3%)	59,319 (42.4%)	14,650 (10.5%)	2024 (1.4%)	<0.001
	Several times a week	16,662 (10.8%)	59,366 (38.5%)	63,857 (41.4%)	13,122 (8.5%)	1313 (0.9%)	
	Once a week	9896 (12.3%)	33,718 (42.0%)	30,515 (38.0%)	5557 (6.9%)	557 (0.7%)	
	A few times a month	25,255 (12.5%)	84,038 (41.6%)	77,863 (38.6%)	13,561 (6.7%)	1193 (0.6%)	
	Once a month	13,426 (14.0%)	40,826 (42.6%)	34,789 (36.3%)	6288 (6.6%)	604 (0.6%)	
	Less than once a month	44,053 (15.0%)	124,916 (42.6%)	104,714 (35.7%)	17,987 (6.1%)	1784 (0.6%)	
	I never drink such drinks	44,915 (0.6%)	96,913 (42.0%)	72,943 (31.6%)	14,079 (6.1%)	1627 (0.7%)	
Energy drinks	Three or more times a day	721 (15.4%)	1199 (25.5%)	1936 (41.3%)	7011 (4.9%)	136 (2.9%)	<0.001
	One to two times a day	1497 (11.5%)	4114 (31.5%)	5714 (43.7%)	1532 (11.7%)	205 (1.6%)	
	Several times a week	4660 (12.7%)	13,064 (35.5%)	15,209 (41.4%)	3355 (9.1%)	480 (1.3%)	
	Once a week	3337 (13.9%)	9675 (40.2%)	8972 (37.3%)	1843 (7.7%)	232 9 (1.0%)	
	A few times a month	7900 (12.7%)	24,119 (38.9%)	24,361 (39.3%)	5108 (8.2%)	571 (0.9%)	
	Once a month	6441 (13.7%)	19,370 (41.1%)	17,515 (37.2%)	3403 (7.2%)	400 (0.8%)	
	Less than once a month	28,204 (14.0%)	83,966 (41.6%)	74,362 (36.8%)	14,025 (6.9%)	1444 (0.7%)	
	I never drink energy drinks	115,856 (14.4%)	333,651 (41.4%)	295,932 (36.7%)	55,277 (6.9%)	5634 (0.7%)	
Drinking alcohol	Two or more drinks a day	3613 (11.2%)	10,552 (32.8%)	13,632 (42.4%)	3809 (11.8%)	562 (1.7%)	<0.001
	About one drink a day	6486 (14.9%)	18,217 (41.9%)	15,899 (36.6%)	2612 (6.0%)	261 (0.6%)	
	Two or three drinks a week	25,548 (15.9%)	70,549 (43.8%)	55,508 (34.4%)	8824 (5.5%)	739 (0.5%)	
	Two or three drinks a month	27,438 (15.0%)	78,640 (43.0%)	65,396 (35.7%)	10,588 (5.8%)	952 (0.5%)	
	One drink a month or less	31,962 (13.5%)	93,046 (39.4%)	91,260 (38.6%)	18,027 (7.6%)	1869 (0.8%)	
	I never drink alcohol	22,551 (13.7%)	56,755 (34.6%)	64,480 (39.3%)	17,730 (10.8%)	2505 (1.5%)	
Vegetables	Every day	78,097 (46.3%)	165,786 (33.9%)	115,349 (26.0%)	20,539 (24.1%)	2227 (24.5%)	<0.001
	Several times a week	63,879 (37.9%)	214,037 (43.8%)	193,689 (43.6%)	34,638 (40.6%)	3208 (35.3%)	
	Once a week	9791 (5.8%)	41,672 (8.5%)	47,044 (10.6%)	9626 (11.3%)	970 (10.7%)	
	A few times a month	11,925 (7.1%)	49,700 (10.2%)	61,554 (13.9%)	12,949 (15.2%)	1480 (16.3%)	
	Once a month	1842 (1.1%)	7635 (1.6%)	10,608 (2.4%)	2823 (3.3%)	379 (4.2%)	
	Less than once month	2283 (4.2%)	8858 (1.8%)	13,326 (3.0%)	3825 (4.5%)	596 (6.5%)	
	Never	793 (0.5%)	1467 (0.3%)	2428 (0.5%)	841 (1.0%)	240 (2.6%)	
Fruits	Every day	78,535 (46.6%)	183,567 (37.5%)	132,318 (29.8%)	22,227 (26.1%)	2325 (25.6%)	<0.001
	Several times a week	59,343 (35.2%)	191,243 (39.1%)	171,329 (38.6%)	30,739 (36.1%)	2914 (32.0%)	
	Once a week	11,505 (6.8%)	43,412 (8.9%)	47,982 (10.8%)	9785 (11.5%)	917 (10.1%)	
	A few times a month	12,559 (7.4%)	49,233 (10.1%)	60,539 (13.6%)	13,133 (15.4%)	1421 (15.6%)	
	Once a month	2505 (1.5%)	9312 (1.9%)	12,735 (2.9%)	3495 (4.1%)	426 (4.7%)	
	Less than once a month	3099 (1.8%)	10,391 (2.1%)	16,083 (3.6%)	4763 (5.6%)	824 (9.1%)	
	Never	1063 (0.6%)	1997 (0.4%)	3013 (0.7%)	1100 (1.3%)	271 (3.0%)	
Red meat	Every day	5728 (5.3%)	15,317 (4.9%)	17,341 (5.9%)	4110 (7.0%)	489 (7.4%)	<0.001
	One to three times a week	42,387 (39.2%)	126,074 (40.7%)	115,470 (39.5%)	21,544 (36.7%)	2181 (33.1%)	
	Once or twice a month	51,598 (47.7%)	143,146 (46.2%)	129,471 (44.3%)	25,550 (43.5%)	2781 (42.2%)	
	Never	8497 (7.9%)	25,170 (8.1%)	30,255 (10.3%)	7572 (12.9%)	1135 (17.2%)	

^1^ *p*-value derived from the chi-square test for independence.

## Data Availability

The data presented in this study are available upon request from the corresponding author.

## Supplementary Materials

The following supporting information can be downloaded at https://www.mdpi.com/article/10.3390/nu17182924/s1. Table S1: STROBE Statement—Checklist of Items for Cross-Sectional Studies; Table S2: English Translation of Questionnaire Items.
