# Supplementary material for: Socio-Demographic Disparities in Diet and Their Association with Physical and Mental Well-Being: Million-Participant Cross-Sectional Study in Poland
Source: Nutrients. 2025 Sep 11;17(18):2924. doi: 10.3390/nu17182924 (PMC12473112; doi:10.3390/nu17182924)
Supplement: Supplementary file 1 [file nutrients-17-02924-s001.zip › nutrients-3838056-supplementary.pdf]

# Supplementary Material

# S1: STROBE Statement—Checklist of Items for Cross-Sectional Studies

**Study Title:** Socio-demographic Disparities in Diet and their Association with Physical and Mental Well-being: Million-Participant Cross-Sectional Study in Poland

| Section/Topic | Item No. | Recommendation | Page No. | Relevant Text from Manuscript |
|---|---|---|---|---|
| **Title and abstract** | 1 | (a) Indicate the study's design with a commonly used term in the title or the abstract | 1 | Title includes "Cross-Sectional Study" |
| | | (b) Provide in the abstract an informative and balanced summary of what was done and what was found | 1 | Comprehensive abstract provided with background, methods, results, and conclusions |
| **Introduction** | | | | |
| Background/rationale | 2 | Explain the scientific background and rationale for the investigation being reported | 1-2 | Comprehensive background on dietary habits, health outcomes, Polish public health context, and evidence gaps |
| Objectives | 3 | State specific objectives, including any prespecified hypotheses | 2 | Main objective stated: "address this evidence gap by leveraging the uniquely large dataset." Main and specific hypotheses provided |
| **Methods** | | | | |

| | | | | |
|---|---|---|---|---|
| Study design | 4 | Present key elements of study design early in the paper | 2 | "This study employed a cross-sectional design, analyzing data collected from the National Poles' Health Test" |
| Setting | 5 | Describe the setting, locations, and relevant dates, including periods of recruitment, exposure, follow-up, and data collection | 2 | Online survey through Medonet platform, Poland nationwide, 2019-2024 data collection period |
| Participants | 6 | (a) Give the eligibility criteria, and the sources and methods of selection of participants | 2 | "Adult (≥18 years old) Polish internet users who voluntarily participated; no other specific exclusion criteria" |
| | | (b) For matched studies, give matching criteria and number of exposed and unexposed | N/A | Not applicable - no matching performed |
| Variables | 7 | Clearly define all outcomes, exposures, predictors, potential confounders, and effect modifiers. Give diagnostic criteria, if applicable | 2-3 | Outcomes (self-rated health), exposures (dietary habits), predictors (socio-demographics) described |
| Data sources/measurement | 8* | For each variable of interest, give sources of data and details of methods of assessment (measurement). Describe comparability of assessment methods if there is more than one group | 2 | Online questionnaire via Medonet, self-reported measures, pilot testing with 100 participants mentioned |

| | | | | |
|---|---|---|---|---|
| Bias | 9 | Describe any efforts to address potential sources of bias | 2, 10 | Anonymous voluntary participation, informed consent mentioned. More comprehensive discussion in limitations section |
| Study size | 10 | Explain how the study size was arrived at | 3 | The study used convenience sampling through voluntary online participation. No formal sample size calculation was performed as the aim was to achieve the largest possible sample through the available platform. |
| Quantitative variables | 11 | Explain how quantitative variables were handled in the analyses. If applicable, describe which groupings were chosen and why | 2-3 | BMI calculation, age groups, frequency categories mentioned |
| Statistical methods | 12 | (a) Describe all statistical methods, including those used to control for confounding | 3 | Chi-square test, Student's t-test, ANOVA, Mann-Whitney U test, Spearman correlation. Software: Statistica 13.3 |
| | | (b) Describe any methods used to examine subgroups and interactions | 3, 6-8 | Comparative analyses by socio-demographic groups described |

| | | | | |
|---|---|---|---|---|
| | | (c) Explain how missing data were addressed | 4 | Complete case analysis was used for all statistical tests. Participants with missing data for specific variables were excluded from relevant analyses. |
| | | (d) Cross-sectional study—If applicable, describe analytical methods taking account of sampling strategy | 3 | Basic analytical approach described |
| | | (e) Describe any sensitivity analyses | 4 | No sensitivity analyses were performed given the descriptive nature of the study. |
| **Results** | | | | |
| Participants | 13* | Report numbers of individuals at each stage of study—eg numbers potentially eligible, examined for eligibility, confirmed eligible, included in the study, completing follow-up, and analysed | 3 | Total participants (1,196,102) |
| Descriptive data | 14* | (a) Give characteristics of study participants (eg demographic, clinical, social) and information on exposures and potential confounders | 3-4 | Comprehensive Table 1 with participant characteristics |
| | | (b) Indicate number of participants with missing data for each variable of interest | 6 | Missing data rates were <2% for all variables. |

| | | | | |
|---|---|---|---|---|
| | | (c) Cross-sectional study—Summarise follow-up time (eg, average and total amount) | N/A | Not applicable for cross-sectional design |
| Outcome data | 15* | Cross-sectional study—Report numbers of outcome events or summary measures | 4-6 | Comprehensive outcome data in Tables 2-4 showing dietary habits and health associations |
| Main results | 16 | (a) Give unadjusted estimates and, if applicable, confounder-adjusted estimates and their precision (eg, 95% confidence interval). Make clear which confounders were adjusted for and why they were included | 4-8 | Percentages and p-values provided |
| | | (b) Report category boundaries when continuous variables were categorized | 4-6 | Frequency categories clearly defined in tables |
| | | (c) If relevant, consider translating estimates of relative risk into absolute risk for a meaningful time period | N/A | Not applicable for this study design |
| Other analyses | 17 | Report other analyses done—eg analyses of subgroups and interactions, and sensitivity analyses | 6-8 | Correlation analyses, subgroup analyses reported |
| **Discussion** | | | | |

| | | | | |
|---|---|---|---|---|
| Key results | 18 | Summarise key results with reference to study objectives | 8-9 | Key findings well summarized in relation to study objectives and hypotheses |
| Limitations | 19 | Discuss limitations of the study, taking into account sources of potential bias or imprecision. Discuss both direction and magnitude of any potential bias | 10 | Comprehensive limitations section addressing selection bias, self-reporting bias, cross-sectional design limitations |
| Interpretation | 20 | Give a cautious overall interpretation of results considering objectives, limitations, multiplicity of analyses, results from similar studies, and other relevant evidence | 8-10 | Thoughtful interpretation considering objectives, limitations, and comparison with existing evidence |
| Generalisability | 21 | Discuss the generalisability (external validity) of the study results | 10 | External validity limitations well discussed, including population representativeness concerns |
| **Other information** | | | | |
| Funding | 22 | Give the source of funding and the role of the funders for the present study and, if applicable, for the original study on which the present article is based | 11 | Funding source provided (Wroclaw Medical University, SUBZ.C290.25.054) |

# S2: English Translation of Questionnaire Items

## National Poles' Health Test - Relevant Questions Translation

This document provides the English translation of all questionnaire items from the National Poles' Health Test that were used in the study "Socio-demographic Disparities in Diet and their Association with Physical and Mental Well-being: Million-Participant Cross-Sectional Study in Poland."

### 1. Socio-demographic and Anthropometric Variables

**Q1. Sex**

- **Original (Polish):** Proszę zaznaczyć swoją płeć:
- **Translation:** Please select your sex:
- **Response options:**
  a. Woman
  b. Man

**Q2. Age**

- **Original (Polish):** Proszę podać swój wiek.
- **Translation:** Please provide your age.
- **Response format:** Open field for two-digit number

**Q3. Education Level**

- **Original (Polish):** Jakie wykształcenie uzyskał/a Pan/Pani do tej pory?
- **Translation:** What is the highest level of education you have completed?
- **Response options:**
  a. Primary
  b. Lower secondary (gimnazjalne)
  c. Vocational
  d. Secondary
  e. Post-secondary
  f. Bachelor's/Engineering degree

g. Master's degree or higher

**Q4. Place of Residence**

- **Original (Polish):** Proszę zaznaczyć wielkość miejscowości, w której Pan/Pani obecnie mieszka.
- **Translation:** Please select the size of the locality where you currently live.
- **Response options:**
    a. Village
    b. City up to 19,000 inhabitants
    c. City 20,000–49,000 inhabitants
    d. City 50,000–99,000 inhabitants
    e. City 100,000–199,000 inhabitants
    f. City 200,000–499,000 inhabitants
    g. City 500,000 or more inhabitants

**Q6. Height**

- **Original (Polish):** Proszę podać swój wzrost w centymetrach.
- **Translation:** Please provide your height in centimeters.
- **Response format:** Open field for three-digit number (min: 100, max: 250)

**Q7. Weight**

- **Original (Polish):** Proszę podać swoją masę ciała (wagę) w kilogramach.
- **Translation:** Please provide your body weight in kilograms.
- **Response format:** Open field for three-digit number (min: 30, max: 299)

## 2. Health Status and Well-being

**Q7C. Level of Health Education**

- **Original (Polish):** Na ile ocenia Pani/Pan poziom edukacji zdrowotnej, którą zdobyła/ł Pani/Pan podczas procesu wychowania, edukacji i pracy zawodowej, by móc prawidłowo zadbać o swoje zdrowie?
- **Translation:** How do you rate the level of health education you acquired during upbringing, education, and professional work to be able to properly care for your health?

- **Response options:**
    a. Insufficient
    b. Low
    c. Average
    d. Good
    e. Very good

**Q8. Self-rated Physical Health**

- **Original (Polish):** Jak ogólnie ocenia Pan/Pani stan swojego zdrowia fizycznego w porównaniu z innymi osobami w Pana/Pani wieku?
- **Translation:** How do you generally rate your physical health compared to other people your age?
- **Response options:**
    a. Very good
    b. Good
    c. Average (neither good nor bad)
    d. Bad
    e. Very bad

**Q9. Self-rated Mental Health**

- **Original (Polish):** Jak ogólnie ocenia Pan/Pani stan swojego zdrowia psychicznego w porównaniu z innymi osobami w Pana/Pani wieku?
- **Translation:** How do you generally rate your mental health compared to other people your age?
- **Response options:**
    a. Very good
    b. Good
    c. Average (neither good nor bad)
    d. Bad
    e. Very bad

**Q11. Diagnosed Chronic Diseases**

- **Original (Polish):** Czy kiedykolwiek zdiagnozowano u Pana/Pani którąś z poniższych chorób/dolegliwości?
- **Translation:** Have you ever been diagnosed with any of the following diseases/conditions?
- **Response format:** Yes/No for each condition
- **Conditions listed:**
  a. Arterial hypertension
  b. Diabetes
  c. Heart diseases (ischemic heart disease, heart valve defect, heart failure, atrial fibrillation)
  d. Chronic obstructive pulmonary disease (COPD)
  e. Allergy or asthma
  f. Depression
  g. Cancer
  h. Joint disease
  i. Neurological disease
  j. COVID-19

**Q30. Perceived Stress Frequency**

- **Original (Polish):** Jak często doświadcza Pan/Pani stresujących sytuacji?
- **Translation:** How often do you experience stressful situations?
- **Response options:**
  a. Daily
  b. Several times a week
  c. Once a week
  d. Several times a month
  e. Once a month
  f. Less than once a month
  g. Never

## 3. Dietary Habits and Consumption Patterns

**Q31. Type of Diet**

- **Original (Polish):** Jakie posiłki spożywa Pan/Pani najczęściej?
- **Translation:** What type of meals do you eat most often?
- **Response options:**
    a. Balanced according to the food pyramid
    b. Vegetarian
    c. Vegan
    d. Meat-based
    e. Gluten-free
    f. Dairy-free
    g. With carbohydrate restriction (no potatoes, rice, pasta)
    h. With reduced sodium (salt) content
    i. Other type of meals
    j. I don't know/hard to say

**Q31aa. Red Meat Consumption** *(Asked only to non-vegetarian/vegan respondents)*

- **Original (Polish):** Jak często jada Pan/Pani czerwone mięso?
- **Translation:** How often do you eat red meat?
- **Response options:**
    a. Daily
    b. One to three times a week
    c. Once or twice a month
    d. Never

**Q33. Fast Food Consumption**

- **Original (Polish):** Jak często jada Pan/Pani produkty typu fast-food (hamburgery, frytki, hot-dogi itp.)?
- **Translation:** How often do you eat fast-food products (hamburgers, fries, hot dogs, etc.)?
- **Response options:**

a. Daily
b. Several times a week
c. Once a week
d. Several times a month
e. Once a month
f. Less than once a month
g. I never eat such products

**Q34. Sweetened Beverage Consumption**

- **Original (Polish):** Jak często pije Pan/Pani napoje słodzone, gazowane lub niegazowane?
- **Translation:** How often do you drink sweetened beverages, carbonated or non-carbonated?
- **Response options:**
    a. Daily
    b. Several times a week
    c. Once a week
    d. Several times a month
    e. Once a month
    f. Less than once a month
    g. I never drink such beverages

**Q35. Energy Drink Consumption**

- **Original (Polish):** Jak często pije Pan/Pani napoje energetyzujące?
- **Translation:** How often do you drink energy drinks?
- **Response options:**
    a. 3 or more times a day
    b. 1 to 2 times a day
    c. Several times a week
    d. Once a week
    e. Several times a month

f. Once a month
g. Less than once a month
h. I never drink energy drinks

**Q37. Vegetable Consumption**

- **Original (Polish):** Jak często jada Pan/Pani warzywa (nie wliczając ziemniaków i soków z koncentratu)?
- **Translation:** How often do you eat vegetables (not including potatoes and juice from concentrate)?
- **Response options:**
  a. Daily
  b. Several times a week
  c. Once a week
  d. Several times a month
  e. Once a month
  f. Less than once a month
  g. I never eat vegetables

**Q38. Fruit Consumption**

- **Original (Polish):** Jak często jada Pan/Pani owoce (nie wliczając soków z koncentratu)?
- **Translation:** How often do you eat fruits (not including juice from concentrate)?
- **Response options:**
  a. Daily
  b. Several times a week
  c. Once a week
  d. Several times a month
  e. Once a month
  f. Less than once a month
  g. I never eat fruits

**Q49A. Alcohol Consumption**

- **Original (Polish):** Ile alkoholu pije Pan/Pani?
- **Translation:** How much alcohol do you drink?
- **Response options:**
    a. Two or more drinks daily
    b. About one drink daily
    c. Two or three drinks a week
    d. Two or three drinks a month
    e. One drink a month or less
    f. I never drink alcohol

**Note:** This translation includes all questionnaire items that were analyzed in the main study. The response categories and numbering correspond to those used in the original Polish questionnaire and subsequent data analysis. Some questions had additional sub-questions or follow-up items that were not included in the main analysis and are therefore not translated here.
